# Identification of Pathogenetic Brain Regions *via* Neuroimaging Data for Diagnosis of Autism Spectrum Disorders

**DOI:** 10.3389/fnins.2022.900330

**Published:** 2022-05-17

**Authors:** Yu Wang, Yu Fu, Xun Luo

**Affiliations:** ^1^Hunan Provincial Key Laboratory of Intelligent Computing and Language Information Processing, Hunan Normal University, Changsha, China; ^2^College of Information Science and Engineering, Hunan Normal University, Changsha, China; ^3^Hunan Xiangjiang Artificial Intelligence Academy, Changsha, China

**Keywords:** autism spectrum disorders, fMRI, pathogenic brain regions identification, disease diagnosis, random SVM cluster

## Abstract

Autism spectrum disorder (ASD) is a kind of neurodevelopmental disorder that often occurs in children and has a hidden onset. Patients usually have lagged development of communication ability and social behavior and thus suffer an unhealthy physical and mental state. Evidence has indicated that diseases related to ASD have commonalities in brain imaging characteristics. This study aims to study the pathogenesis of ASD based on brain imaging data to locate the ASD-related brain regions. Specifically, we collected the functional magnetic resonance image data of 479 patients with ASD and 478 normal subjects matched in age and gender and used a machine-learning framework named random support vector machine cluster to extract distinctive brain regions from the preprocessed data. According to the experimental results, compared with other existing approaches, the method used in this study can more accurately distinguish patients from normal individuals based on brain imaging data. At the same time, this study found that the development of ASD was highly correlated with certain brain regions, e.g., lingual gyrus, superior frontal gyrus, medial gyrus, insular lobe, and olfactory cortex. This study explores the effectiveness of a novel machine-learning approach in the study of ASD brain imaging and provides a reference brain area for the medical research and clinical treatment of ASD.

## Introduction

Autism spectrum disorder (ASD) is a kind of brain developmental disorder with complex etiology and hidden onset (Lord et al., 2018). It is most often diagnosed in teenagers and children because of the high plasticity of their brain function. Children with ASD will suffer various difficulties in early development, including slow response to sensory information (e.g., hearing, smell, and taste), lagged language learning, limited interest, difficulty interacting with others, etc. (Vallianatos et al., 2018; McKinnon et al., 2019; Kang et al., 2020; Santore et al., 2020). Currently, there is no specific therapy for ASD in the clinic, which will cause the long-term economic burden of family and social support (Helkkula et al., 2020). Considering that the ages of patients are relatively small, it is difficult to diagnose based on the general quantitative evaluation of social behavior in clinics. Therefore, looking for characteristic biomarkers to help clinical workers make accurate clinical decisions in the early stage is an important research direction at present (Frye et al., 2019).

Magnetic resonance imaging (MRI) is a commonly applied technique for diagnosing brain diseases in clinical, which can intuitively show the location and degree of brain lesions. MRI is currently playing a major auxiliary role in the treatment and research of complex brain diseases, such as ASD (Hao et al., 2017; Du et al., 2019; Dryburgh et al., 2020; Yang et al., 2021). Specifically, functional MRI (fMRI) is a new neuroimaging technique that can measure the hemodynamic changes caused by neuronal activity and generate a time series to reflect the activity characteristics of certain brain regions. Some open-access datasets, such as autism brain imaging data exchange (ABIDE), usually contain sufficient fMRI data, which greatly promotes the development of relevant research (Di Martino et al., 2014, 2017). For example, Cheng et al. (2017) conducted a knowledge-based enrichment analysis of fMRI data of patients with autism and healthy controls (HCs) and found that some functional connections (FCs) decreased significantly at the network circuit level. Through specific correlation analysis technology, the correlation networks among multiple regions of interest (ROIs) can be established, which provides a broader perspective in pathogenetic studies (Franzmeier et al., 2019; Noble et al., 2019). For example, Ingalhalikar et al. (2021) obtained fMRI data in ABIDE, proposed a novel technology to eliminate differences between sites, and found several important FCs of patients with ASD.

Efficiency is usually an important factor in imaging data analysis. In recent years, machine-learning algorithms have been increasingly used in dimension reduction and feature extraction in brain imaging data and have played a key role in the research of ASD and many other brain diseases (Abraham et al., 2017; Heinsfeld et al., 2018; Li et al., 2020). Among the existing machine-learning approaches, a support vector machine (SVM) can keep a stable performance in optimizing the feature dimension of samples (Guo et al., 2019; Wei et al., 2019). In the research related to ASD, Chaitra et al. (2020) proposed a new feature-eliminating mechanism to iteratively improve the classification ability of the trivial SVM method and obtained the connected feature subset with better ASD recognition ability. Osredkar et al. (2019) combined SVM and radial kernel function with 4 urine biomarkers to diagnose ASD and found that the levels of 8-hydroxy-2′-deoxyguanosine and 8-isoproterenol in urine can improve the diagnosis performance.

The extraction of the most discriminative features is the central work to ensure the efficiency of SVM. However, most previous studies focused on optimizing single SVM classifiers. The disadvantage of such approaches is that current methods of feature extraction can hardly avoid remaining some important features incorrectly ignored and the screened features can difficult be reconsidered after the optimization of a single SVM classifier. Also, the pathogeny identification in the existing studies is often based on limited data, which may lead to few reliable results and weak generality. Therefore, this study applies a random SVM cluster framework to extract features from fMRI data to classify patients with ASD and HCs (Bi et al., 2018). With the help of ensemble learning, only the features shared by most classifiers are extracted as the important features, which reduces the blindness in feature selection and prevents over-fitting even under a large scale of data. As the results indicate, the optimized random SVM cluster performs well in classifying patients with ASD and HCs, and the feature extraction results are consistent with many existing studies. Compared to the other existing works, this study provides an attractive framework to detect the disease-associated factors of ASD based on the fMRI data.

## Materials and Methods

### Overview

**Figure 1:**
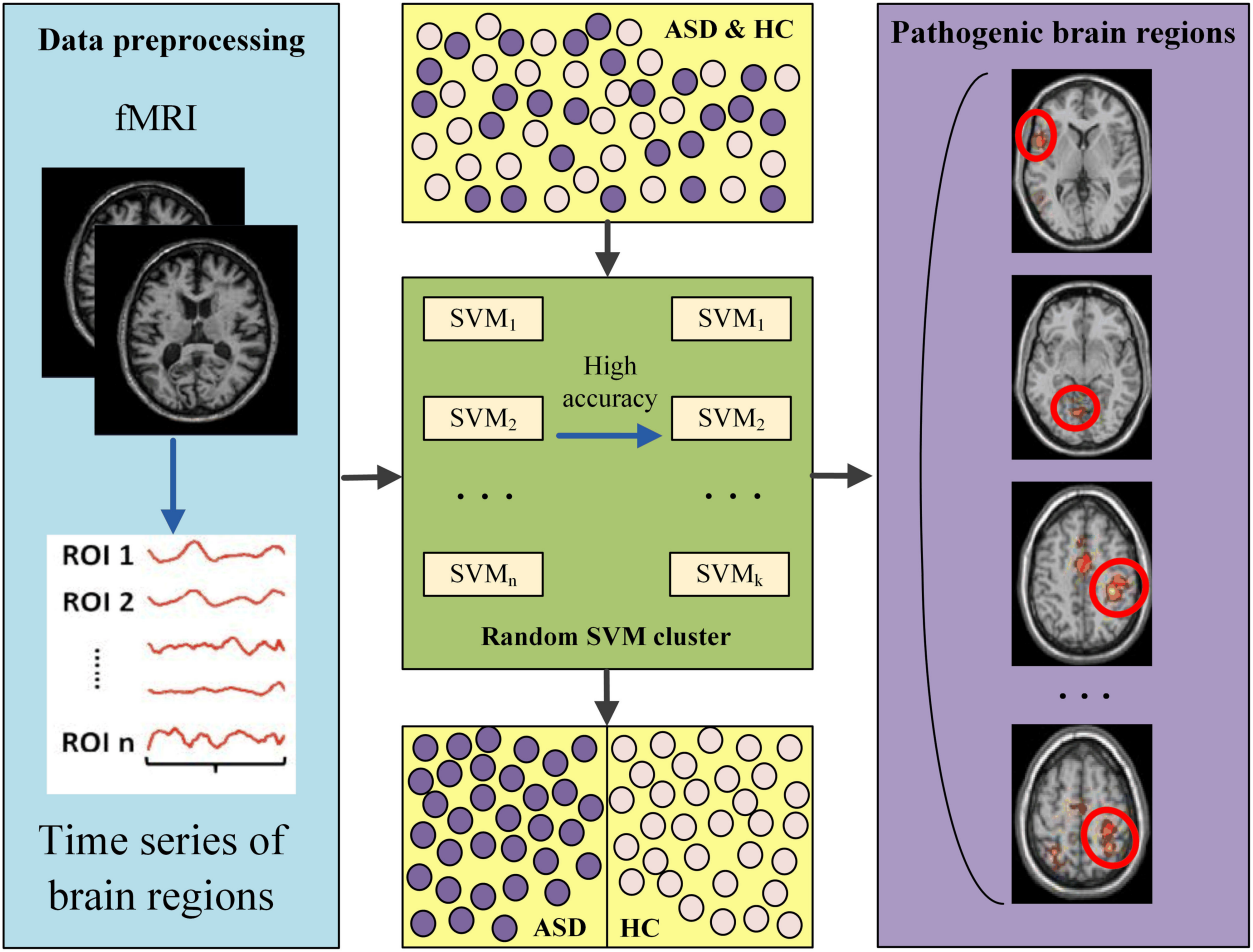
The entire workflow of this study.

The entire analysis pipeline of this study can be divided into three major parts, which are depicted in Figure 1. First, the fMRI data are preprocessed, resulting in the time series for each ROI. Second, a random SVM cluster is constructed to extract the characteristic features. Finally, further analysis is conducted to identify pathogenetic brain regions.

### Subjects

All biological data in this study are from the ABIDE database and do not involve bio-standard safety measures and institutional safety procedures. The data acquirement has been approved by relevant departments and complies with relevant standards. The subjects used in this study are determined through further screening. This study has tried to keep as many samples as possible to ensure the robustness of the conclusion. However, it is a necessity to eliminate the data that occur errors while preprocessing. At last, it remains raw fMRI data of 479 patients with ASD and 478 HCs obtained from ABIDE-I for analysis. All HCs have signed written consent and are out of any other neurological diseases. ABIDE database has strict standards for data collection and processing, which ensures the homology of data structure.

The online datasets extendedly provide additional information on subjects, including age, full-scale intelligence quotient (FIQ), performance intelligence quotient (PIQ), and verbal intelligence quotient (VIQ). The latter three indicators are used to quantify the comprehensive performance of the intellectual function. This study evaluated the statistical differences in the above attributions between ASD and HC groups. The basic information of the two groups of subjects is shown in Table 1, which indicates no significant difference in all indicators among the participants.

**Table 1:** Basic information of the subjects.

**Variable**	**Patients with ASD**	**HC subjects**	***p*** **-value**
Age	16.70 ± 8.23	17.20 ± 8.06	0.798^*^
FIQ	105.21 ± 16.56	111.20 ± 12.80	0.000^**^
PIQ	104.89 ± 17.06	108.61 ± 13.31	0.001^**^
VIQ	103.25 ± 18.05	110.37 ± 13.50	0.000^**^

* *This study calculated the p-value corresponding to the age through chi-square test*.

** *This study calculated the p-values corresponding to FIQ, PIQ, and VIQ using two-sample t-test. All information listed in this table is expressed by the format of “mean ± standard deviation.” It shows no statistical difference between two groups of data if the corresponding p-value is >0.05*.

### Data Preprocessing

All fMRI data are collected from MRI scanners whereas the subjects are in the resting state, which means all subjects are relaxed without doing any thinking work during the scanning. To conduct data preprocessing, this study uses a Data Processing Assistant for Resting-State fMRI (DPARSF),^1^ a widely applied tool in the MATLAB platform that is dedicated to fMRI preprocessing (Karpiel et al., 2019). Specific steps of preprocessing are listed as follows:

(1) Inputting the raw DCM files and converting the data format to NIFTI;
(2) Deleting the initial 10 time points and slice timing;
(3) Realigning the head movement to eliminate artifact effect;
(4) Readjusting, including standardizing the functional image to echo plane imaging template and smoothing;
(5) Eliminating the residual noise which increases or decreases over time;
(6) Temporal filtering to maintain the fluctuation being within 0.01 ± 0.08 Hz;
(7) Removing covariates and head movements that may affect unnatural BOLD fluctuations.

### Construction of Sample Features

Functional connections can reflect the organization and interrelationships among different ROIs even if they are not histologically connected. In this study, FCs are constructed as the features of the samples. Specifically, the weight value of an FC is calculated to represent the tightness of the corresponding ROI–ROI pair. Concrete construction steps are as follows.

(1) Separating all brain images into ROIs according to the automatic anatomical labeling template, which is applied in many fMRI-based studies (Liu et al., 2016). The applied template can generate 116 ROIs in total, of which 90 ROIs belong to the brain whereas the other 26 ROIs belong to the cerebellum. As a common practice, this study focuses only on the brain and the ROIs in the cerebellum are therefore omitted.
(2) Calculating the Pearson correlation coefficient value between each ROI pair as the FCs. Before the calculation, time series are normalized and further sliced to a uniform length to eliminate the effect of inter-site difference (Esteban et al., 2017; Wang et al., 2022). The higher the coefficient value, the stronger the FC between two ROIs.
(3) Constructing sample features. For each sample, a vector composed of 4,005 (i.e., the number of free combinations among 90 ROIs) weight values of FCs is calculated as the sample feature for subsequent experiments. In other words, the dimensionality of the original sample features is 4,005 in this study.

### Construction of the Random Support Vector Machine Cluster

In machine learning, excessively big feature dimensionality may cause mass computing load. However, if the feature dimensionality is too small, a large amount of important information may be lost. Ensemble learning is an effective strategy that can effectively improve the model performance by integrating single classifier to form clusters and generate results through a voting mechanism (Chen et al., 2018a; Wei et al., 2018). According to our previous research, ensemble learning shows great potential in feature selection (Bi et al., 2020, 2021). In this study, this study adopted a method named random SVM cluster to effectively analyze the high-dimensional data, which has performed well in the fMRI-based study of Alzheimer's disease. The concrete procedure details are as follows.

First, the initial sample set *S* is divided into three subsets, namely, a training set *S*_1_ with 382 subjects, a verifying set *S*_2_ with 96 subjects, and a testing set *S*_3_ with 479 subjects. The size rate is ~4:1:5. The proportion balance of patients and HCs is kept during the division.

Second, to construct an SVM classifier, *M* samples are randomly selected from the training set *S*_1_.and a *d*-dimension sub-feature is generated by randomly selecting *d* components from the original 4,005-dimension feature. The above procedure improves the diversity of SVM classifiers, which, according to the theory of ensemble learning, will bring significant improvement to the generalization performance of the integrated learner. By repeating the above procedure for *n* times, *n* SVMs are derived and the random SVM cluster is constructed accordingly.

Third, the SVM classifiers are further screened for optimization. Specifically, the verification set *S*_2_ is applied to evaluate all constructed SVM classifiers by their respective classification accuracies. The classifiers with classification accuracies lower than 0.5 will be deleted, in that such performance is inferior to the randomly guessing and will passively affect the performance of the overall cluster. After the selection, *k* superior SVMs (*k*< *n*) have been selected to form a new cluster.

Finally, sample classification and feature extraction are conducted using the random SVM cluster. Concretely, *k* screened SVMs in the cluster separately classify the samples in the testing set *S*_3_ and generate the final result through the majority voting mechanism. By calculating the ratio of the number of correctly classified samples to the size of the *S*_3_, the accuracy of the entire cluster is obtained.

### Identification of Pathological Brain Regions

The SVM classifiers in this study are generated through the random selection of features and samples, which makes the features of each classifier not the same. At the same time, the classification accuracy of a classifier indicates the significance of its corresponding features. After the screening of classifiers, the remaining features are taken as the important features that have strong classification ability for ASD and HC. In other words, ASD and HC have more obvious differences in these characteristics, which means that the ROIs contained in these characteristics are more prone to functional or structural damage. Further, considering that these features are defined as FCs of ROI–ROI pairs, the ROIs that appear the most in the important features are selected as the pathological brain regions. The specific procedure of pathogeny identification is as follows.

(1) Sorting and determining the superior classifiers. All SVM classifiers are sorted in descending order of classification accuracy. Then, a classifier whose accuracy is greater than a certain threshold of 0.75 would be determined as the superior classifier.
(2) Extracting the optimal features. The appearance frequency of each feature in superior SVM classifiers is calculated. Then, the features with the highest frequencies are extracted as the optimal features, which represent the most discriminative FCs between patients with ASD and HC subjects.
(3) Determining the pathological brain regions of ASD. In this study, this study defined the weight of an ROI as its appearance frequency in optimal features. The ROIs with the highest weights are taken as the pathological brain regions.

## Results

### Performance Comparison With Existing Methods

**Figure 2:**
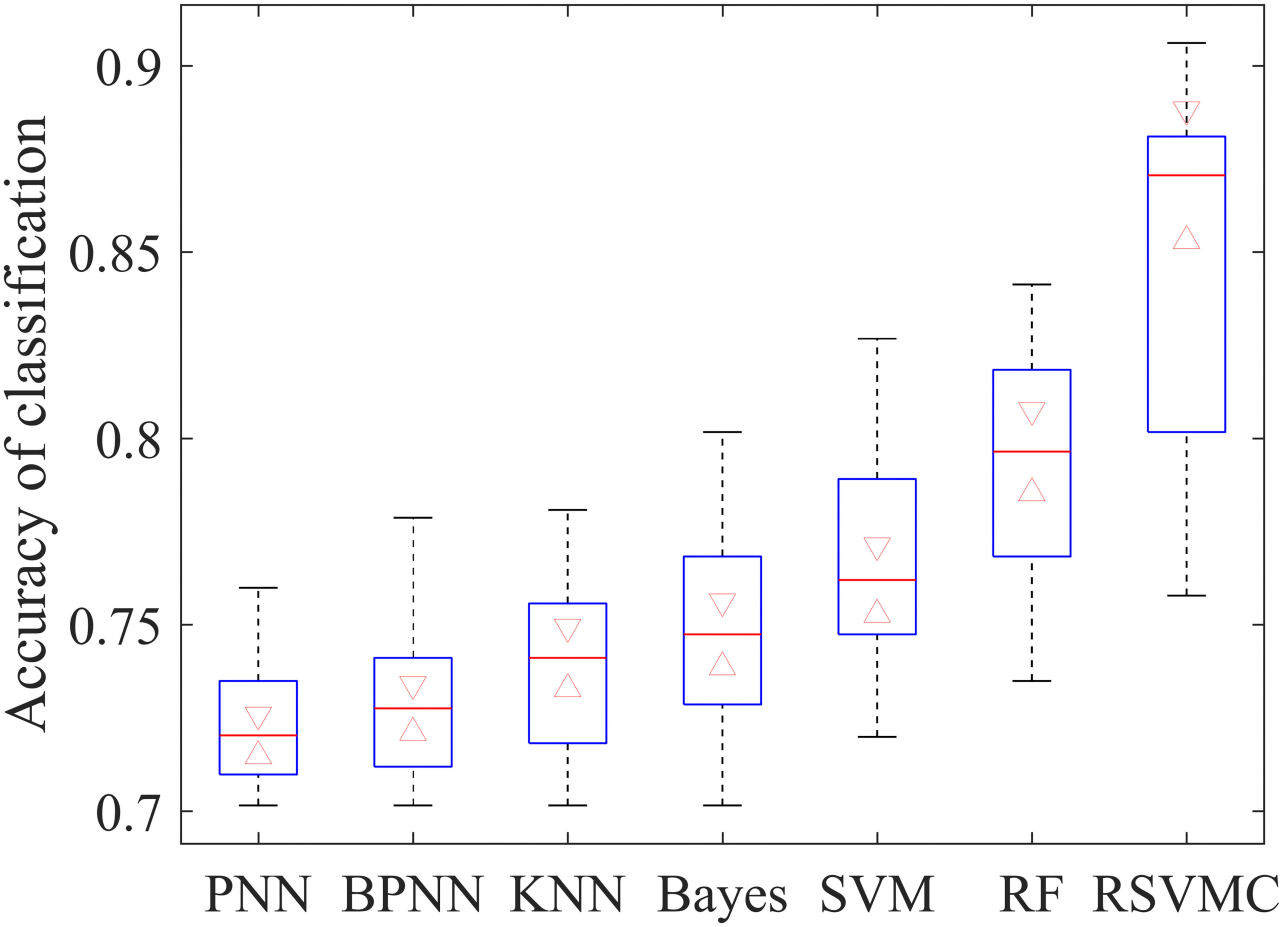
Classification accuracy of different classification method.

To certify the efficiency of the random SVM cluster, this article compared its performance with other common approaches for feature selection. The baseline methods include product-based neural network (PNN), backpropagation neural network (BPNN), K-nearest neighbor (KNN), naïve Bayes classifier (Bayes), single SVM classifier (SVM), random forest (RF), and random SVM cluster (RSVMC). Considering the randomness in sample division and feature selection, all comparative methods have been repeated 50 times to avoid accidental errors. The box plot in Figure 2 depicts the comparative results.

It could be observed that the applied random SVM cluster was significantly superior to the baseline approaches. It is worth noting that the random forest method, as a typical ensemble learner, performed better than all single learners, which indicates the effectiveness of ensemble learning. Also, the highest accuracy of single SVM among all single learners proves the superiority of SVM in brain imaging analysis. Such results implied that the superiority of our method derives from the effective integration of the advantages of SVM and ensemble learning.

**Figure 3:**
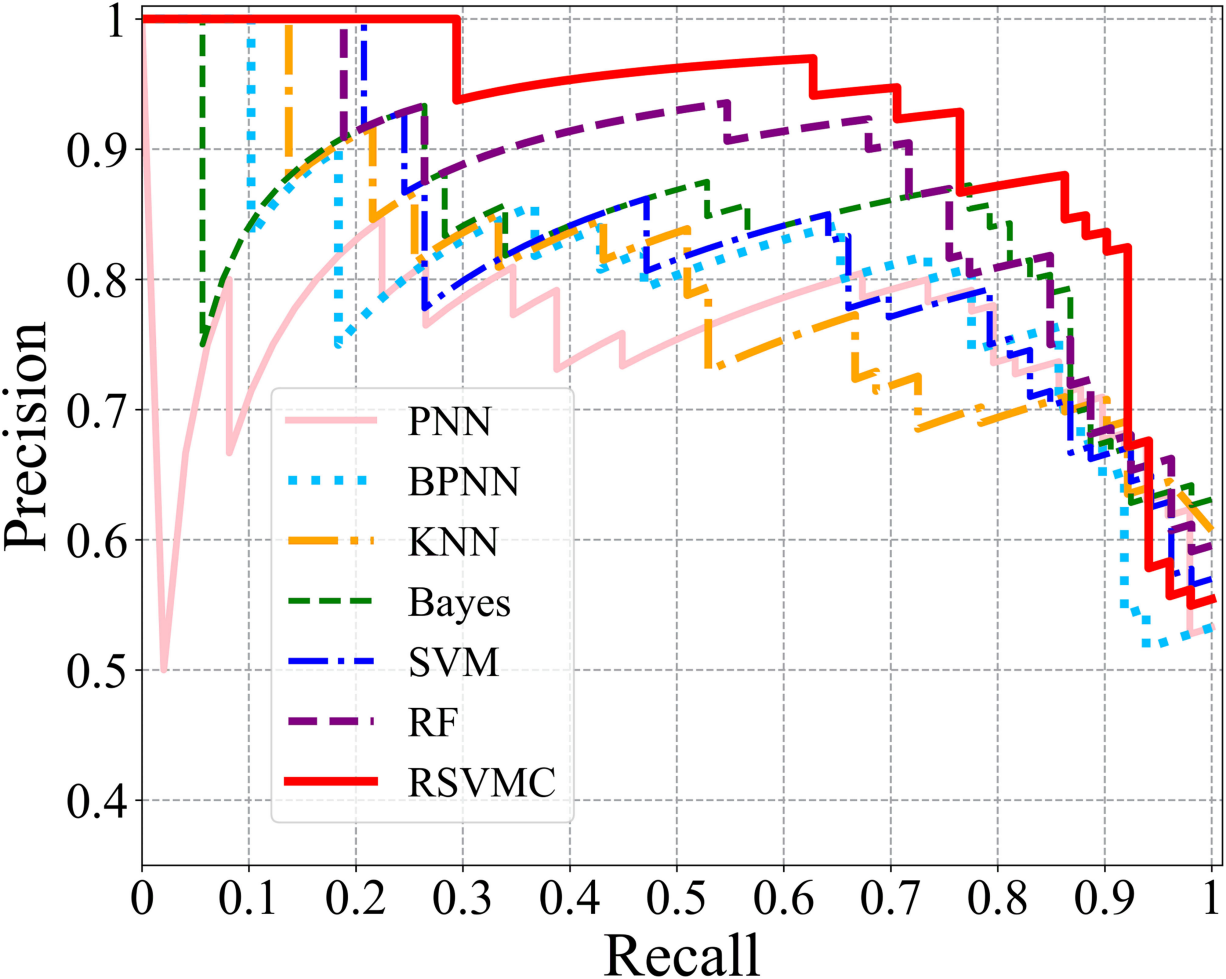
The P-R curves of comparative methods.

To compare the performances of the machine-learning methods, this study further calculated the precision and recall values of all comparative methods. Figure 3 depicted the Precision-Recall (P-R) curves of all competing methods, where we could observe that the random SVM cluster owned the highest position among all methods, and the superiority of our method is confirmed from another angle.

### Parameter Optimization

According to the method definition, the SVM number *n* and feature dimensionality *d* are the two important parameters to be optimized, which is critical to finalizing a well-performed random SVM cluster. Parameter optimizing results are shown as follows.

**Figure 4:**
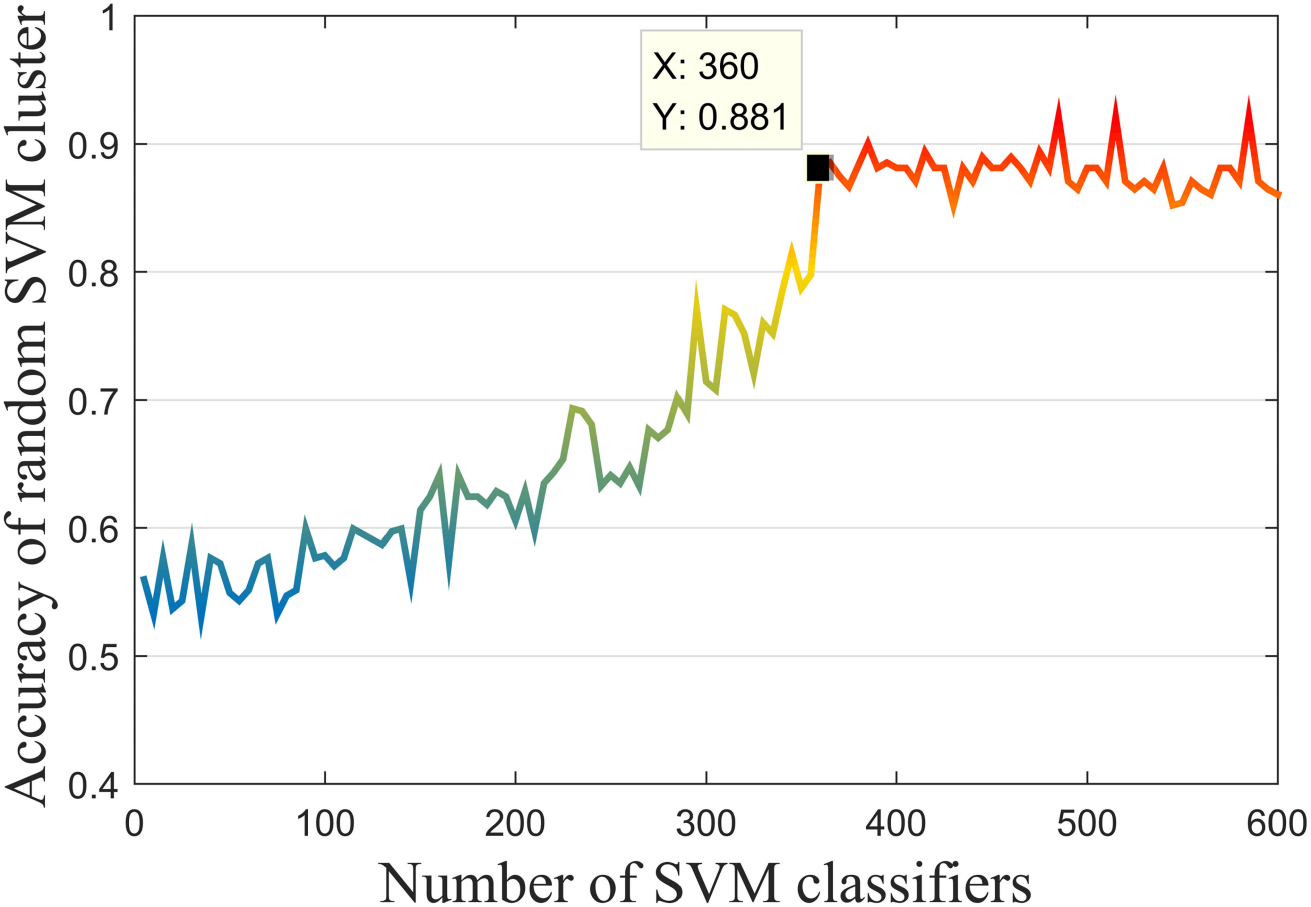
Performance of the random SVM cluster with different number of SVM.

On the one hand, this study conducted experiments to find the optimal number of SVM classifiers in the initial cluster. Specifically, the number of the SVM was gradually increased from 5 to 600 with a step length of 5, during which the accuracy of the random SVM cluster had first increased and then tended to be stable. According to the experimental results depicted in Figure 4, it can be observed that when the cluster includes 360 SVMs, the overall performance started to be stable. Thus, the base classifier number *n* was determined as 360.

**Figure 5:**
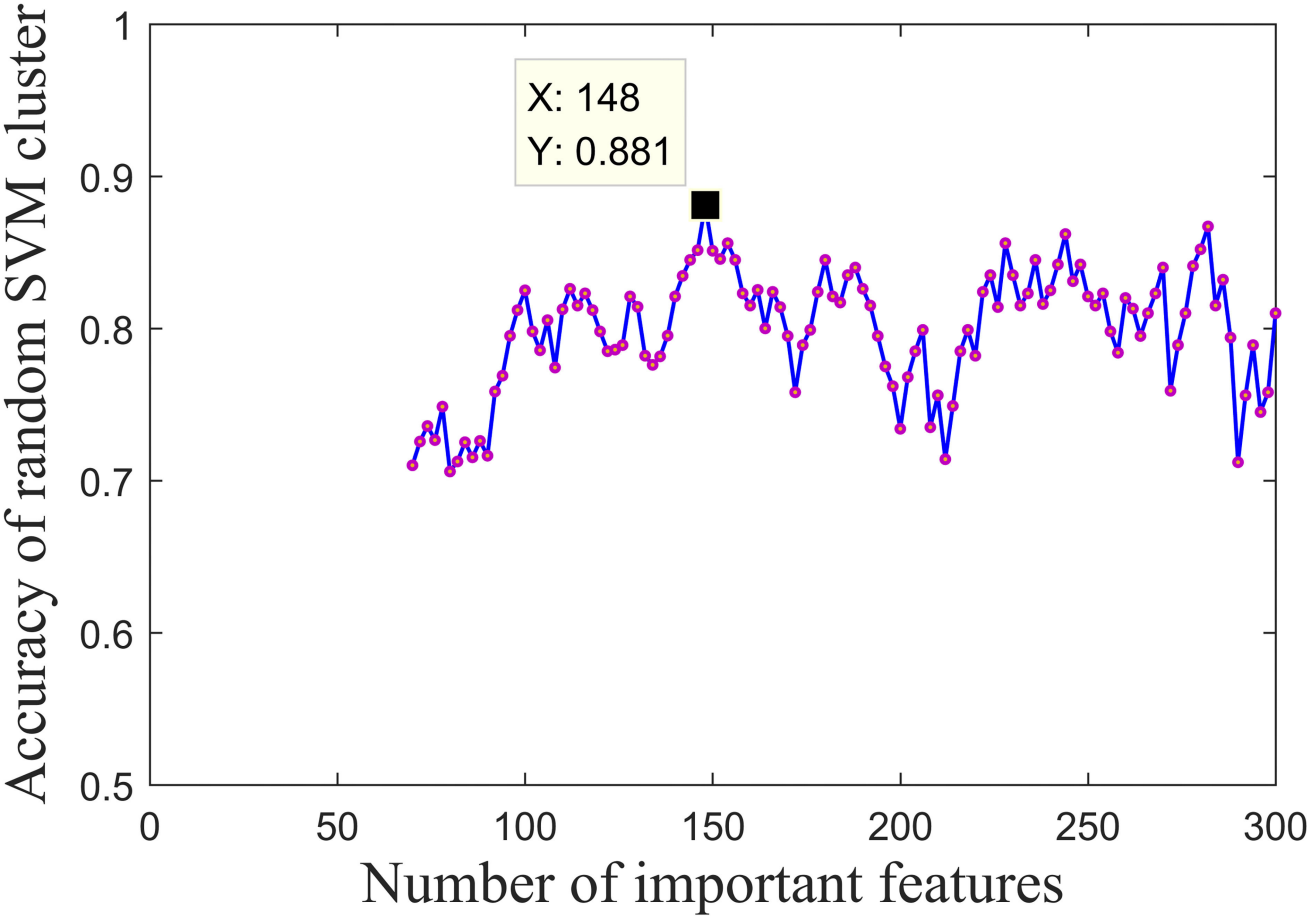
Accuracies of the random SVM cluster with different numbers of important fusion features.

On the other hand, this study determined the optimal feature dimensionality. To ensure the performance of the model, the conventional practice is to set the feature dimensionality as the square root of the original feature dimensionality (Belgiu and Drǎgu, 2016). However, considering the data complexity of fMRI, such means may cause a great loss of important information, which inspired us to expand and optimize the feature dimensionality. First, this study built the initial cluster with 70 out of 4,005 randomly opted features. The initial number is determined as 70 because it was an approximate value of the square root of 4,005. Subsequently, this study increased the feature dimensionality up to 300 in a step of 2 and calculated the overall accuracy of the cluster in each iteration. Finally, this study took the feature dimensionality corresponding to the highest accuracy as the optimal dimensionality of features. Figure 5 depicted the results during all iterations, which showed that when the feature dimensionality is determined as 148, the accuracy was 88.1%. It is worth noting that the equivalence of accuracies in two optimization experiments was an accident and these two experiments were carried out successively, which means that in the second experiment, the number of SVM classifiers was determined as 360 according to the former experiment.

### Extraction Results of the Pathological Brain Regions

**Figure 6:**
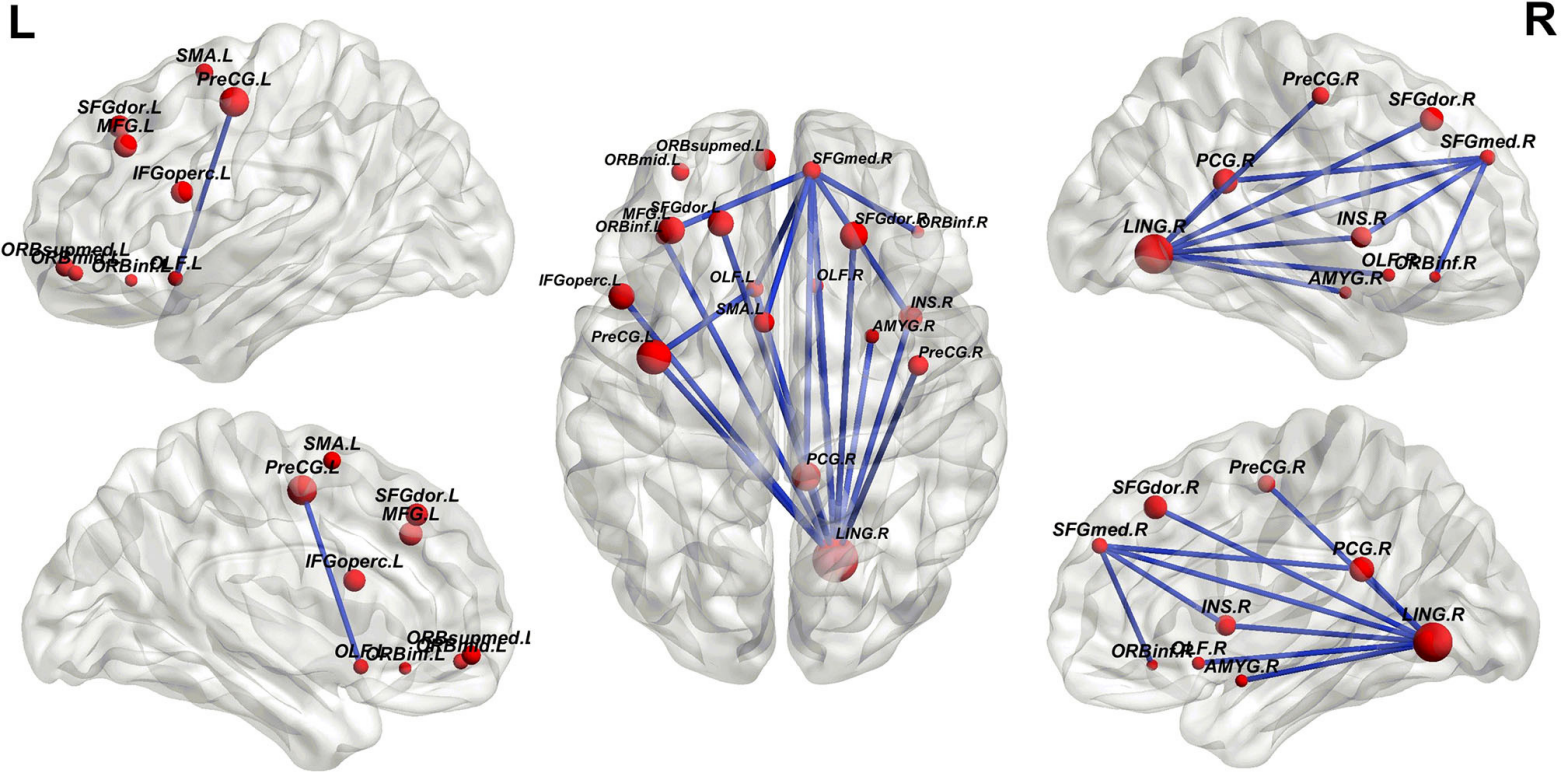
Top 20 functional connections corresponding to the extracted optimal features.

**Figure 7:**
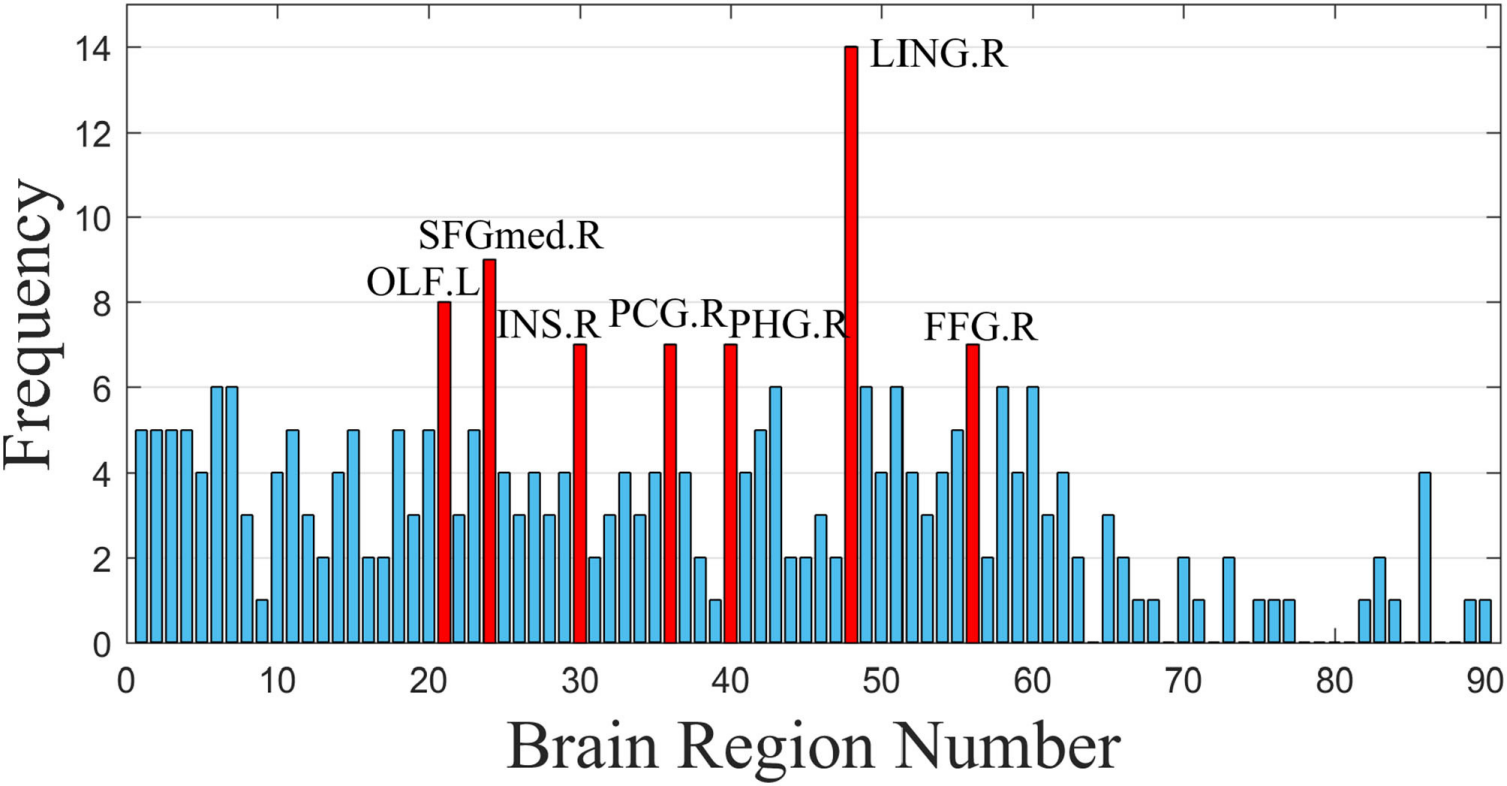
Frequencies of all ROIs.

By fitting the optimal number of features to 148, the performance of the random SVM cluster was maximized and the cost of computing resources was concurrently reduced. Consequently, 148 discriminative FCs in patients with ASD and HCs were obtained. The top 20 FCs with the highest frequencies were visualized in Figure 6, where the node size that corresponds to each brain region represents the weight of the brain region, that is, the frequency of the brain area. The larger the node, the higher the frequency of brain regions. Subsequently, the frequencies of all 90 ROIs included in the 148 FCs are shown in Figure 7.

## Discussion

This study utilized an improved SVM learner and achieved the classification accuracy of 88.1% in patients with ASD identification. Compared with other recent endeavors based on ABIDE datasets, our method also shows superiority in classification performance. Liu et al. (2020) proposed a multi-task objective function to extract the dynamic functional connectivity specific to ASD, archiving an accuracy of 76.8%. Wang et al. proposed a new method integrating ensemble learning with sparseness constraints and tested the method on two different sites of data in ABIDE, obtaining accuracies of 72.6 and 71.4%, respectively. Epalle et al. (2021) improved the deep neural network model and tested the proposed classification framework based on cross-validation, achieving the final accuracy value of 78.07%.

As shown in Figure 7, the discriminative FCs of ASD mainly existed in the lingual gyrus (LING.R), superior frontal gyrus, medial (SFGmed.R), olfactory cortex (OLF.L), insula (INS.R), parahippocampal gyrus (PHG.R), posterior cingulate gyrus (PCG.R), and fusiform gyrus (FFG.R). On the one hand, our findings were consistent with other existing studies of single brain regions. For instance, Herringshaw et al. (2016) utilized meta-analysis methods to quantify the common and consistent brain activation patterns that usually develop the language processing control, and the results showed that the activation of the LING.R in patients with ASD increased. Lee et al. (2020) used a univariate universal linear model to determine the regions of average connectivity differences between male and female subjects with ASD, and the results showed similar gender differences in the tongue gyrus and the posterior cingulate gyrus cortex. Qian et al. (2018) analyzed the time-varying connectivity using the resting-state fMRI data to investigate brain state mutations in children with ASD and finally found abnormal connectivity between INS.R and visual network (and in the middle). Glerean et al. (2016) calculated the correlation between the hemodynamic time courses of each pair of 6-mm isotropic voxels and the proportional inclusiveness between all pairs of subjects, and the results indicated that the subjects who had lower autism quotient scores conversely showed significantly higher nodule intensity in certain brain regions, including FFG.R.

On the other hand, some connection-based studies also verify the results in this article. Huang et al. (2019) enhanced the representation of FC networks by fusing and conveying the public and supplementary information into multiple networks to identify the biomarkers of neuropsychiatric diseases, and the results indicated that OLF.L in subcortical regions is a potential discriminative brain region. Liu and Huang (2020) applied multivariate model analysis to study the connectedness subset of whole-brain FC, finding out that the severity of ASD with SFGmed.R and SFGmed.L changed significantly. Noriega (2019) adopted a sliding-time window method based on an adjusted time span to study whether the time proportion of correlation measure was above or below the average, and the results showed that the FC related to OLF.L was significantly enhanced in controls relative to ASD-severe. Delbruck et al. studied the action observation network of children with ASD and observed that atypical connectomes related to FFG.R showed great significance to the social cognitive defection.

Some other highly-rated ROIs found in our work, such as the PCG.R and PHG.R, were rarely studied in other research about ASD. Nevertheless, certain studies have indicated their potential relation to ASD. For example, PCG.R has been presently found as the tissue correlated with sensation, stereo location, and memory. The hippocampus is an emotion regulation center, which had been long paid special attention in depression research. In addition, as the main cortex of the hippocampus, PHG.R can significantly affect the cognitive and emotional functions of the brain. Thus, the findings in this article may provide a new insight for further exploration of the pathological mechanism of ASD.

Despite the satisfactory performance, our study still has limitations. First, in this study, this study uses a normalization strategy to preprocess fMRI data obtained from multiple sites, whereas many advanced approaches have been proposed to eliminate the inter-site differences (Moradi et al., 2017; Wang et al., 2019), which may be applied in future work. Second, this study utilizes the Anatomical Automatic Labeling (AAL) template for brain segmentation, but there are many other proposed templates, e.g., the Harvard-Oxford Atlas template, which may provide some quite different information and help to discover different types of FCs (Lei et al., 2020). Finally, in this study, this study only analyzed the medical imaging data for feature extraction. In the follow-up work, we will try to expand the data types in various ways and may involve genes, cells, electrocardiographs, or other clinical phenotypes for further research (Raka et al., 2017; Wang et al., 2017; Chen et al., 2018b; Du et al., 2020).

## Conclusion

This article conducted an fMRI-based study for ASD diagnosis using a machine-learning approach named random SVM cluster. Defining the sample features as FCs among ROIs, the pathological factors of ASD were explored. According to the experimental results, discriminative ROIs of patients with ASD and HCs were identified, including LING.R, SFGmed.R, OLF.L, INS.R, PCG.R, PHG.R, and FFG.R. The contributions of our work can be summarized in two key points. On the one hand, an efficient random SVM cluster was applied for ASD diagnosis. On the other hand, some pathological FCs and ROIs highly related to the development of ASD are identified, which can provide valuable references for the medical research and clinical treatment of ASD.

## Data Availability Statement

Publicly available datasets were analyzed in this study. This data can be found here: https://ida.loni.usc.edu/login.jsp?project=ABIDE.

## Ethics Statement

This study was carried out in accordance with the recommendations of National Institute of Aging-Alzheimer's Association (NIA-AA) Workgroup Guidelines. The study was approved by Institutional Review Board (IRB) of each participating site, including the Banner Alzheimer's Institute, and was conducted in accordance with Federal Regulations, the Internal Conference on Harmonization (ICH), and Good Clinical Practices (GCP). The patients/participants provided their written informed consent to participate in this study.

## Author Contributions

YF proposed the design of the work and revised it critically for important intellectual content. XL organized the original draft of the paper and drafted part of the work. YW collected, interpreted the data, and drafted part of the work. YF and XL carried out the experiment for the work. All authors contributed to the article and approved the submitted version.

## Funding

This work was supported by the National Natural Science Foundation of China (62072173), Natural Science Foundation of Hunan Province, China (2020JJ4432), Key Scientific Research Projects of Department of Education of Hunan Province (20A296), Key Open Project of Key Laboratory of Data Science and Intelligence Education (Hainan Normal University), Ministry of Education (DSIE202101), National Key Research and Development Program of China (2020YFB2104400), the Hunan Provincial Science and Technology Project Foundation (2018TP1018), Medical Humanities and Social Sciences Project of Hunan Normal University, and Innovation & Entrepreneurship Training Program of Hunan Xiangjiang Artificial Intelligence Academy.

## Conflict of Interest

The authors declare that the research was conducted in the absence of any commercial or financial relationships that could be construed as a potential conflict of interest.

## Publisher's Note

All claims expressed in this article are solely those of the authors and do not necessarily represent those of their affiliated organizations, or those of the publisher, the editors and the reviewers. Any product that may be evaluated in this article, or claim that may be made by its manufacturer, is not guaranteed or endorsed by the publisher.

## Footnotes

^1^ http://rfmri.org/dpabi
